# Investigation of nutrition status and analysis of 180-day readmission factors in elderly hospitalized patients with COPD

**DOI:** 10.1007/s40520-024-02820-9

**Published:** 2024-08-01

**Authors:** Huan Liu, Jingsi Song, Zhiqiang Wang, Songze Wu, Shi Qiu, Benhui Chen, Zhiyong Rao, Xiaofan Jing

**Affiliations:** 1 Department of Clinical Nutrition, West China Hospital, Sichuan University, Chengdu, China; 2 Department of Clinical Nutrition, Chengdu Shang jin Nan fu Hospital, West China Hospital, Chengdu, China; 3 Department of Respiratory and Critical care Medicine, West China Hospital, Sichuan University, Chengdu, China; 4 Department of Medical administration, West China Hospital, Sichuan University, Chengdu, China; 5 Department of Integrated Chinese and Western Medicine, West China Hospital, Sichuan University, Chengdu, China

**Keywords:** COPD, GLIM, Malnutrition, 180-day readmission rate, Elderly

## Abstract

**Background and objective:**

Malnutrition is prevalent among elderly patients with COPD, who also experience a high rate of readmission. Therefore, it is imperative to investigate the nutrition status of these patients, identify risk factors for readmission, and offer insights for clinical management. To achieve this, a cross-sectional study was conducted to investigate factors influencing nutrition status using GLIM criteria and explore the 180-day readmission factors among hospitalized elderly COPD patients.

**Methords and results:**

The data were collected from a hospital in Southwest China, encompassing a cohort of 319 eligible patients. Among elderly hospitalized COPD patients, the prevalence of malnutrition was 49.53% (158/319). Multivariate logistic regression revealed malnutrition (OR = 3.184), very severe airway obstruction (OR = 3.735), and Number of comorbidities ≥ 3 (OR = 5.754) as significant risk factors for 180-day readmission.

**Conclusions:**

These findings suggest that malnutrition is a prevalent issue among elderly hospitalized patients with COPD and constitutes one of the risk factors contributing to the 180-day readmission rate. Therefore, timely identification and treatment for malnourished patients are crucial.

## Introduction

Chronic obstructive pulmonary disease (COPD) is a heterogeneous lung condition characterized by chronic respiratory symptoms, such as dyspnea, cough, sputum production and/or exacerbations.These symptoms arise due to abnormalities in the airways (bronchitis, bronchiolitis) and/or alveoli(emphysema) leading to persistent, often progressive, airflow obstruction [1]. The latest report from the World Health Organization(WHO) revealed that COPD is currently one of the top three leading causes of death worldwide, accounting for 6% of all deaths [2]. The prevalence of COPD increases sharply with age, peaking among individuals aged 60 and above [1]. In China, the prevalence of COPD is 13.7% among adults aged 40 and above, while it exceeds 27% among those aged 60 and above [3].

Researchers in related fields are increasingly focusing on the comorbidities of COPD; however, nutrition-related comorbidities, especially malnutrition, are often overlooked [4]. Factors such as increased resting energy expenditure, inflammation, hypoxia, and medication use contribute to the high incidence of malnutrition among patients with COPD, potentially impacting disease progression, particularly in the elderly [5]. Malnutrition is closely associated with poor clinical outcomes and imposes a significant burden on healthcare resources [6]. It has been reported that 20-45% of hospitalized COPD patients suffer from malnutrition, depending on the population and diagnostic methods used [7]. To establish a global consensus on the clinical diagnosis of malnutrition, the Global Leadership Initiative on Malnutrition (GLIM) criteria have been published [8]. The Chinese Society for Parenteral and Enteral Nutrition (CSPEN) notes that the GLIM criteria are suitable for Chinese patients and can be effectively used to diagnose malnutrition [9].

Additionally, there are currently few studies using the GLIM criteria to evaluate the nutrition status of elderly hospitalized COPD patients; most research focuses on patients in communities or rehabilitation centers [10–15]. COPD is a globally prevalent disease with a high readmission rate [16]. It remains unclear whether nutrition status affects the readmission rate of COPD patients; further analysis of the factors influencing this rate is needed. Therefore, this study aims to evaluate the nutrition status of elderly hospitalized COPD patients using the GLIM criteria, determine the incidence of malnutrition, and explore the relevant factors that affect the readmission rate. The ultimate goal is to reduce the readmission rate and provide a reference for future clinical treatment.

## Materials and methods

### Design

The study is a cross-sectional analysis conducted in accordance with the Strengthening the Reporting of Observational Studies in Epidemiology recommendations [17]. The Biomedical Ethics Review Committee of West China Hospital, Sichuan University, approved this study (Approval No. 1080).

### Setting

The study was conducted in the Department of Respiratory and Critical Care Medicine at a university hospital in southwestern China, spanning from March 2021 to September 2022. In accordance with the hospital’s stringent management protocols, all admitted patients were required to undergo COVID-19 testing. Patients who tested positive were transferred to the Infectious Disease Department.

### Participants

**Fig. 1:**
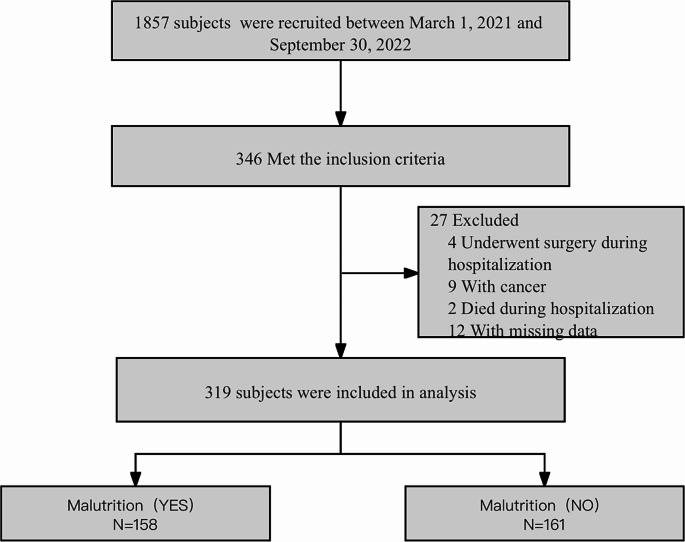
Flow chart of the patient selection process

The study included patients admitted to the Department of Respiratory and Critical Care Medicine with a primary diagnosis of COPD, according to the GOLD 2021 guidelines [18], aged 65 years or elder, and admitted for the first time due to COPD. Following the application of the inclusion and exclusion criteria, 319 patients were included in the analysis. For additional details, refer to Fig. 1.

### Study variables

According to the GLIM criteria, the primary outcome variables include malnutrition, hospital readmission within 180 days post-discharge, length of hospital stay, and hospitalization costs, all obtained from electronic medical records. For the analysis, we established a dichotomous variable for nutritional status (malnutrition vs. non-malnutrition).

The GLIM criteria was assessed as follows:

- Nutritional Screening: The Nutritional Risk Screening 2002 (NRS 2002), a forward-looking and clinically validated tool, serves as the first step in the diagnosis of malnutrition according to the GLIM criteria [19]. Scores of 3 or above are indicative of nutritional risk [20], while scores below 3 indicate no nutritional risk.
- Diagnosis of malnutrition: For hospitalized elderly patients at nutritional risk, the GLIM criteria are used for nutritional assessment. According to the GLIM criteria [8], the diagnosis of malnutrition requires the presence of at least one phenotypic criterion and one etiologic criterion.
- Phenotypic criteria: 1)Involuntary weight loss: >5% over 180 days or > 10% over a period longer than 180 days [8];2)low BMI: for Asian populations, < 18.5 kg/m² for individuals under 70 years old, and < 20.0 kg/m² for those over 70 years old [8]; 3)muscle depletion: currently, normal values for muscle mass indicators and clinically supported “cutoff points” are lacking in China. The GLIM criteria recommend using indicators such as calf circumference (CC) and arm muscle circumference (AMC) as alternative methods for muscle mass assessment when body composition measurements are not feasible [8]. The European Working Group on Sarcopenia in Older People (EWGSOP) also considers calf circumference as an indicator of muscle mass in elderly individuals when other muscle mass assessment methods are not feasible. Thus, calf circumference (≤ 30 cm for men and ≤ 29 cm for women) is used as a substitute [21].
- Etiologic criteria: (1) reduced food intake or impaired digestion and absorption; (2) disease burden/inflammatory state: COPD patients typically exhibit chronic disease-related inflammatory responses, which align with the criteria for mild to moderate inflammation in the GLIM guidelines. Besides demographic characteristics (age, sex, ethnicity, BMI, and smoking history), the study considered laboratory values (hemoglobin, blood urea nitrogen, creatinine, total protein, and albumin), comorbidities (number and specific conditions such as hypertension, diabetes mellitus, coronary heart disease, ischemic heart disease, heart failure, atrial fibrillation, osteoporosis, depression/anxiety, gastroesophageal reflux, and bronchiectasis), as well as outcomes (total hospital cost and length of stay).

And the other variables were collected:

e. Evaluate the daily living activities’ independence of elderly patients using the Barthel Index (BI).
f. Assess the severity of airflow obstruction based on the Global Initiative for Obstructive Lung Disease guidelines [18].

### Ethics

This observational study received approval from the Biomedical Ethics Review Committee of West China Hospital, Sichuan University (2020 Audit No. 1080).

### Statistical analysis

Study subject characteristics were summarized as follows: categorical variables were presented as absolute numbers and percentages; normally distributed variables as mean ± standard deviation (SD); and skewed variables as median with interquartile range (IQR). Normality assumptions were assessed using the Kolmogorov‒Smirnov test. Bivariate analyses employed chi-square tests, Mann–Whitney U tests, and Student’s t-tests for paired and independent samples, as appropriate. Mean differences for continuous variables were reported with 95% confidence intervals (95% CI). Multiple logistic regression was conducted to explore the risk of readmission among elderly COPD patients hospitalized for 180 days, with model fit evaluated using the Hosmer–Lemeshow test. Receiver operating characteristic (ROC) curves assessed the predictive performance of the model for the 180-day readmission rate, providing metrics such as area under the curve (AUC), sensitivity, and specificity. Statistical significance was set at *p* < 0.05.

## Results

Based on the inclusion and exclusion criteria, 319 patients were included in the analysis. Table 1 presents the baseline characteristics of the patients: 209 (65.52%) males and 110 (34.48%) females, with a median age of 76 years (IQR: 70–82). Nearly 60% of the subjects had severe (33.54%) or very severe (26.33%) airflow obstruction. The median number of comorbidities was 5 (range:3–7).According to the GLIM criteria, 83 (49.53%) patients were considered malnourished. Regarding smoking history, 40.75% were never smokers, 32.29% were current smokers, and 26.96% were previous smokers. The median length of stay was 12 days, and the total hospital cost was 13,714.78 yuan. Table 1 summarizes the baseline characteristics of the participants.

**Table 1:** Patient’s baseline characteristics (*n* = 319)

	Total (*n* = 319)
**Demographic**	
Sex	
Female, n (%)	110(34.48)
Male, n (%)	209(65.52)
Age, median [IQR](years)	76 [70,82]
Ethnicity	
Han, n (%)	313 (98.12)
Zang, n (%)	5 (1.57)
Qiang, n (%)	1(0.31)
Married	
YES, n (%)	247(77.4)
NO*, n (%)	72(22.6)
Barthel Index (Self-care Ability, score)	
Worse, ≤ 20, n (%)	30(9.41)
Poor, 21–40, n (%)	35(10.97)
Acceptable, 41–60, n (%)	172(53.91)
Good, ≥ 60, n (%)	82(25.71)
BMI, median [IQR](kg/m^2^)	20.88 [18.47,23.88]
**Emergency Admission**	
Yes, n (%)	102(31.97)
No, n (%)	217(68.03)
**Clinical characteristics**	
Severity of airflow obstruction	
Moderate, n (%)	128(40.13)
Severe, n (%)	107(33.54)
Very Severe, n (%)	84(26.33)
Malnutrition	
Yes, n (%)	158(49.53)
No, n (%)	161(50.47)
**Comorbidities**	
Number Of Comorbidities, median [IQR]	5[3,7]
Hypertension, n (%)	111(34.8)
Diabetes Mellitus, n (%)	64(20.06)
Coronary Heart disease, n (%)	37(11.6)
Ischemic Heart disease, n (%)	2(0.63)
Heart Failure, n (%)	11(3.45)
Atrial Fibrillation, n (%)	28(8.78)
Osteoporosis, n (%)	16(5.02)
Depression/Anxiety, n (%)	6(1.88)
Gastroesophageal reflux, n (%)	11(3.45)
Bronchiectasis, n (%)	38(11.91)
**Smoking history**	
Never, n (%)	130(40.75)
Current, n (%)	103(32.29)
Previous, n (%)	86(26.96)
**Laboratory Values**	
HB (g/L)	126.71(SD 22.24)
Lymphocyte (10^9^/L)	1.15(SD 0.63)
BUN (mmol/L)	6.84(SD 2.87)
CREA (mmol/L)	82.90(SD 28.87)
TP (g/L)	62.77(SD 6.66)
ALB (g/L)	37.00(SD 4.56)
**Outcomes**	
Total Hospital Cost, median [IQR] (yuan)	13,714.78[9715.51,18637.55]
Length Of Stay, median [IQR] (days)	12[9,15]

SD: standard deviation; BMI: body mass index; NO *: spouse died or divorced. HB: hemoglobin;

BUN: blood urea nitrogen; CREA: creatinine; TP: total protein; ALB: albumin.

Table 2 describes the main characteristics of patients with and without malnutrition according to the GLIM criteria. Patients without malnutrition (the “NO” group) exhibited significantly better laboratory values of HB, lymphocytes, TP, and ALB compared to those with malnutrition. Furthermore, the total hospital costs of were higher in the “NO” group than in the “YES” group. The “YES” group also had a longer length of hospital stay. Moreover, the 180-day readmission rate was significantly lower in the “NO” group compared to the “YES” group (10.56% vs. 30.38%, *p* < 0.001).

**Table 2:** Group Characteristics of the Laboratory Values and outcome

Variables	Malnutrition		Mean differences (95%CI)	Z-Value	χ2	*p*-Value
	YES(*n* = 158)	NO(*n* = 161)				
**Laboratory Values**					-	
HB (g/L)	122.03(SD 21.95)	131.31(SD 21.62)	-9.28(-14.078 to -4.480)	-	-	< 0.001
Lymphocyte (10^9^/L)	1.06(SD 0.61)	1.24(SD 0.65)	0.178(0.391 to 0.316)	-	-	0.007
TP (g/L)	61.86(SD 6.09)	63.67(SD 7.07)	-1.81(-3.266 to -0.355)	-	-	0.015
ALB (g/L)	36.26(SD 4.52)	37.74(SD 4.48)	-1.47(-2.47 to -0.48)	-	-	0.004
**Outcomes**						
Total Hospital Cost, median [IQR] (yuan)	14,423.59 [10,485.99, 20,878.19]	12,482.8 [8966.32, 16,608.9]	-	-3.001	-	0.003
Length Of Stay, median [IQR] (days)	13[10,15.25]	11[8,14]	-	-2.330	-	0.020
180-day readmission rate, n (%)	48(30.38%)	17(10.56%)	-	-	19.309	< 0.001

Logistic regression analysis was performed to identify the risk factors associated with the 180-day readmission rate. All potential risk factors were included as independent variables, and a logistic model was constructed with the 180-day readmission as the dependent variable (Table 3). The results indicated that malnutrition (OR = 3.184, *p* < 0.001, 95% CI: 1.687–6.009), having three or more comorbidities (OR = 5.754, *p* = 0.021, 95% CI: 1.305–25.379), and very severe airway obstruction (OR = 3.735, *p* < 0.001, 95% CI: 1.784–7.822) were significant risk factors for readmission within the subsequent 180 days (Table 4). The goodness-of-fit test demonstrated that the model had an excellent fit (*p* = 0.938).

**Table 3:** Results of the multivariate logistic regression analysis

Factor	B	SE	Wald χ2	*p*-Value	OR (95%CI)
Malnutrition	1.307	0.309	17.86	0.000	3.696(2.016,6.668)
Age(years)	-0.002	0.019	0.008	0.931	0.998(0.962,1.036)
**BMI**					
<20 kg/m^2^			13.945	0.001	
20 kg/m^2^ ≤ ~ 27 kg/m^2^	-1.086	0.301	12.983	0.000	0.338(0.187,0.609)
≥ 27 kg/m^2^	-0.981	0.573	2.935	0.087	0.375(0.122,1.152)
Number Of Comorbidities^a^	1.712	0.739	5.362	0.021	5.542(1.301,23.609)
Length Of Stay(days)	0.042	0.027	2.538	0.111	1.043(0.990,1.099)
**Ethnicity**					
Han			1.109	0.574	
Zang	0.973	0.924	1.109	0.292	2.646(0,433,16.172)
Qiang	-19.825	40192.970	0.000	1	0.000
Married	-0.113	0.174	0.427	0.514	0.893(0.635,1.255)
**Barthel Index Score**					
≤ 20			0.181	0.981	
21–40	-0.060	0.333	0.032	0.858	0.942(0.491,1.809)
41–60	0.125	0.486	0.066	0.797	1.133(0.437,2.937)
≥ 60	-0.045	0.532	0.007	0.932	0.956(0.337,2.709)
Diabetes Mellitus	-0.542	0.390	1.932	0.165	0.581(0.271,1,249)
Coronary heart disease	0.260	0.411	0.400	0.527	1.297(0.579,2.904)
Hypertension	-0.140	0.296	0.223	0.637	0.870(0.487.1.553)
Ischemic Heart disease	0.188	1.425	0.017	0.895	1.207(0.074,19.720)
Heart Failure	0.599	0.637	0.883	0.347	1.820(0.522,6.342)
Atrial Fibrillation	-0.295	0.399	0.544	0.461	0.745(0.340,1.630)
Osteoporosis	-0.517	0.529	0.957	0.328	0.596(0.211,1.681)
Depression/Anxiety	0.019	0.824	0.001	0.981	1.019(0.203,5.128)
Gastroesophageal reflux	-0.994	0.687	2.096	0.148	0.370(0.096,1.422)
Bronchiectasis	-0.340	0.350	0.945	0.331	0.712(0.359,1.412)
Emergency Admission	0.351	0.312	1.264	0.261	1.420(0.770,2.628)
HB^b^ value(g/L)	-0.464	0.288	2.598	0.107	0.628(0.357,1.106)
Lymphocyte^c^ value(10^9^/L)	-0.232	0.283	0.672	0.412	0.793(0.456,1.381)
TP^d^ value(g/L)	0.152	0.286	0.275	0.600	1.164(0.660,2.052)
ALB^e^ value(g/L)	-0.539	0.349	2.382	0.123	0.584(0.295,1.156)
Never Smoking			1.192	0.551	
Current Smoking	-0.304	0.336	0.818	0.366	0.738(0.381,1.426)
Previous Smoking	-0.316	0.336	0.885	0.347	0.729(0.377,1.409)
**Severity of airflow obstruction**					
Moderate			24.056	0.000	
Severe	0.499	0.383	1.693	0.193	1.647(0.777,3.491)
Very Severe	1.662	0.361	21.225	0.000	5.269(2.598,10.685)

(a) Number of comorbidities ≥ 3; (b) HB value ≥ 120 g/L; (c) lymphocyte value *n* ≥ 1.1*109/L; (d) TP value ≥ 65 g/L; (e) ALB value ≥ 40 g/L.

**Table 4:** Results of multivariate logistic regression analysis

Factor	B	SE	Wald χ2	*p*-Value	OR (95%CI)
Malnutrition	1.158	0.324	12.771	0.000	3.184(1.687,6.009)
Number of comorbidities ≥ 3	1.750	0.757	5.341	0.021	5.754(1.305,25.379)
Severity of airflow obstruction					
Moderate			14.494	0.001	
Severe	0.310	0.395	0.617	0.432	1.364(0.629,2.958)
Very Severe	1.318	0.377	12.212	0.000	3.735(1.784,7.822)

**Table 5:** Results of Hosmer-Lemeshow test

χ2	Sig.	*p*-Value
1.274	5	0.938

**Fig. 2:**
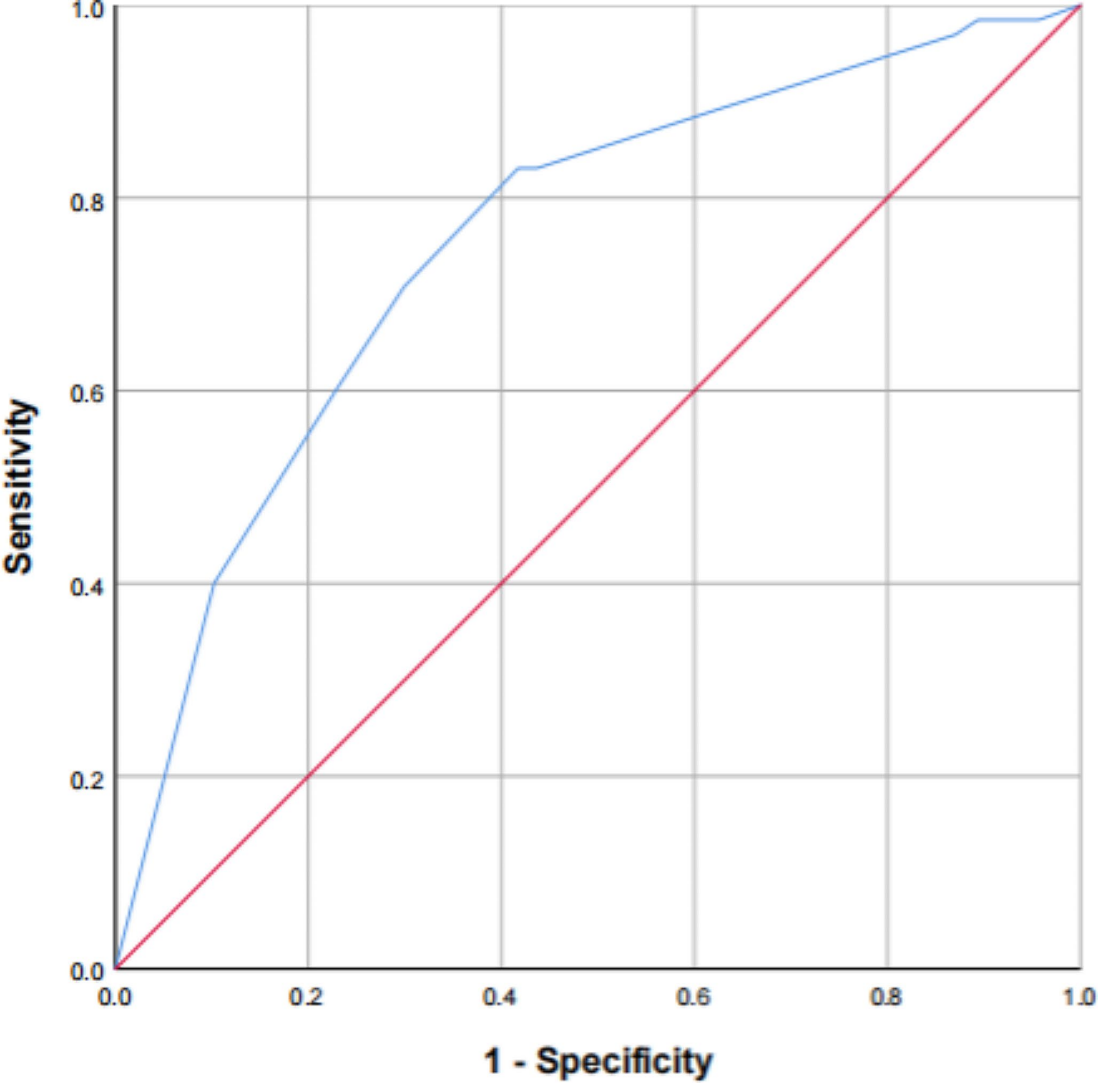
ROC curve prediction for 180-day readmission rate

The area under the Receiver Operating Characteristic (ROC) curve was 0.755(95% CI: 0.689, 0.821), indicating good discriminative ability as illustrated in the column chart. Readmission withinthe subsequent 180 days was a significant predictor (*p* < 0.001), as depicted in Fig. 2 and Table 6.

**Table 6:** Results of the ROC analysis

Factor	AUC	95%CI	SE	*p*-Value	Sensitivity	Specificity
Malnutrition + Number of comorbidities ≥ 3 + Severity of airflow obstruction	0.755	0.689,0.821	0.034	0.000	83.1%	58.3%

## Discussion

The GLIM criteria diagnose malnutrition based on meeting one phenotype and one etiological type [8]. COPD, an inflammatory condition [18], fulfills one of the etiological criteria for malnutrition according to GLIM. Elderly patients with COPD are particularly susceptible to muscle atrophy, weight loss, or low body weight due to inflammation and increased energy consumption, resulting in pathological indicators [10, 22, 23]. The GLIM criteria offer advantages over other nutritional evaluation tools, including more comprehensive assessment, higher malnutrition detection rates, and greater operability [24–27].

In our study, involving 319 elderly COPD patients admitted to the respiratory department, we found that nearly half presented with malnutrition upon admission. This prevalence exceeded that reported by Kaluźniak et al. (22.6%) [10], possibly due to variations in patient populations (rehabilitation centers vs. hospitals) and the severity of illness observed in our study population. Moreover, malnutrition can impair immune function and respiratory muscle performance, significantly affecting patients’ quality of life [28]. Nutrition therapy is crucial in COPD management, as recognized by the European Respiratory Society [29]. Early identification and intervention for malnutrition are essential to alleviate healthcare burdens among elderly adults [30], particularly those with COPD.

The parameters HB, TB, and ALB are recognized as pivotal indicators of nutrition status in COPD patients [31]. Our study found that patients with malnutrition had significantly lower parameters, compared to non-malnutrition individuals. The observed decline in these parameters can be attributed to the heightened metabolic demands and reduced dietary intake commonly associated with COPD, thereby exacerbating the overall deterioration of the nutrition status.

One of the findings of our study is that patients suffering from malnutrition have lower lymphocyte counts. Lymphocytes play a crucial role in the body’s immune response [32]. A systematic review revealed that malnutrition can lead to immunological alterations, and impaired immune function is associated with poorer clinical outcomes [33]. Additionally, compared to non-malnourished individuals, malnourished patients experienced longer hospital stays and higher rates of readmission within 180 days, consistent with the findings of numerous studies [34–37]. Consequently, preventing malnutrition is crucial for improving clinical outcomes.

COPD places a substantial burden on patients, healthcare systems, and society [38, 39]. Patients with COPD often experience exacerbations that usually require hospitalization and readmission [40], further exacerbating the socioeconomic burden. GOLD suggests that COPD patients are often prone to comorbidities such as cardiovascular disease and malnutrition, which can affect their readmission rates [41]. Additionally, the readmission rate is related to multiple factors. The results of this study indicate that malnutrition, very severe airway obstruction, and having three or more comorbidities are significant risk factors for 180-day readmission in elderly COPD patients. A multicenter study reported that the readmission rate of COPD patients within 180 days ranged from 17.9 to 63.0% [40]. This study showed that the readmission rate of elderly hospitalized COPD patients was 20.38%, potentially related to factors such as comorbidities, post-discharge disease management, and the socioeconomic status of the study subjects [40, 42]. Additionally, this study further analyzed the number of comorbidities and revealed that having three or more comorbidities is a risk factor for 180-day readmission in elderly COPD patients. Therefore, for patients with multiple comorbidities, it is crucial to manage and treat these conditions to reduce the risk of readmission in elderly COPD patients.

Marco E et al. reported that patients with COPD are prone to malnutrition [7], which is also a predictive factor for their readmission [43]. This study yielded similar results. With advancing age, bodily functions deteriorate, and digestive and chewing abilities decrease, leading to a higher incidence of malnutrition in elderly patients. Concurrently, muscle attenuation weakens respiratory muscle strength, further impairing respiratory function and promoting acute exacerbations of COPD [44]. Malnutrition is identified as one of the modifiable factors in several high-quality studies related to readmission. Nutritional intervention can increase lean body mass in malnourished COPD patients, enhance their respiratory muscle function, and improve their overall health status [45]. Therefore, active nutritional interventions should be implemented for patients with malnutrition.

Interestingly, this study also revealed that very severe airway obstruction increased the 180-day readmission rate, while severe and moderate airway obstruction had no significant impact. Research has shown that lesions in the small airways and destruction of the lung parenchyma cause airflow limitation. In chronic inflammation, fibrotic repair in the airways leads to fibrosis and narrowing, promoting airflow limitation [46]. For elderly COPD patients with very severe airway obstruction, pulmonary rehabilitation education and pulmonary function exercises should be strengthened, and medications should be regularly used to improve or maintain pulmonary function and reduce readmission [47]. Additionally, other studies have shown that active pulmonary rehabilitation exercises within one month after discharge can significantly reduce the readmission rate and mortality in COPD patients [48].

However, this study has several limitations. First, this was a cross-sectional study, so causal relationships among the factors could not be determined. Second, the research subjects were elderly hospitalized patients with COPD. Whether the findings are generalizable to elderly patients in the community or those in rehabilitation hospitals needs further verification. Finally, due to the lack of a gold standard for evaluating muscle mass reduction, grip strength was used as an alternative assessment based on the GLIM criteria.

## Conclusions

According to the GLIM criteria, the prevalence of malnutrition in elderly hospitalized patients with COPD was high (49.53%). Regression analysis revealed that malnutrition is a risk factor for 180-day readmission in elderly patients with COPD. Therefore, it is crucial to monitor the nutritional status of hospitalized elderly patients with COPD, identify malnourished patients promptly, and actively improve their nutritional status to reduce adverse clinical outcomes.

## Data Availability

No datasets were generated or analysed during the current study.
